# Epidemiological profile and renal outcomes in a 25-year Brazilian cohort of glomerular diseases

**DOI:** 10.1590/2175-8239-JBN-2024-0213en

**Published:** 2025-08-15

**Authors:** Fernando Sales, Priscylla Vieira do Carmo, Rosália Maria Nunes Henriques Huaira, Nicolas William Gonçalves de Almeida, Mateus Henrique Toledo Lourenço, Natália Maria da Silva Fernandes

**Affiliations:** 1 Universidade Federal de Juíz de Fora, Hospital Universitário, Juiz de Fora, MG, Brazil.

**Keywords:** Cohort Studies, Renal Insufficiency, Chronic, Epidemiology, Glomerulonephritis, Pathology

## Abstract

**Introduction:**

Glomerular diseases (GD) are an important cause of chronic kidney disease (CKD). This study aims to analyze the socio-demographic, clinical, and renal outcome profiles of patients with GD.

**Methods:**

A retrospective cohort study was conducted between 1998 and 2023. Participants were patients aged ≥ 18 years, diagnosed with GD, and treated at a university hospital in Brazil. Socio-demographic, clinical, histopathological, and kidney variables were analyzed. A comparative analysis was performed among the most frequent histopathological diagnoses. Renal survival was the outcome variable.

**Results:**

We evaluated 417 patients, of whom 57.3% were women and 69.8% were white. The mean age was 41.7 ± 14.4 years. Primary glomerular diseases (PGD) accounted for 51.1%, and 77.5% of patients underwent a kidney biopsy. The most frequent PGD was membranous nephropathy (26.3%), while among the secondary glomerular diseases (SGD), lupus nephritis (LN) was the most common (51.9%). Minimal change disease was associated with better renal survival, whereas membranoproliferative glomerulonephritis had the worst outcomes (p = 0.001). Regarding CKD progression, a higher initial estimated glomerular filtration rate (eGFR) was protective (HR = 0.956, 95%CI: 0.920–0.994; p = 0.023), while the presence of interstitial fibrosis/tubular atrophy (IFTA) (HR = 1.079, 95%CI: 1.020–1.142; p = 0.008) and a higher body mass index (BMI) (HR = 1.257, 95%CI: 1.016–1.556; p = 0.035) were associated with increased risk of CKD progression.

**Conclusion:**

LN remained the most common SGD in our region, while focal segmental glomerulosclerosis was the third most common PGD. Risk factors for worse outcome included a higher BMI, lower eGFR, and higher IFTA.

## Introduction

Chronic kidney disease (CKD) is considered one of the major public health challenges worldwide and an independent risk factor for cardiovascular diseases (CVD)^1,2^. The estimated global prevalence of CKD across all five stages is 13.4% (11.7–15.1%), affecting more than 800 million people^3,4^. In Brazil, the national prevalence of chronic dialysis patients increased from 665 per million people in 2019 to 758 in 2022^5^.

Glomerular diseases (GD) are the third leading cause of dialytic CKD in Brazil and several other countries, following systemic arterial hypertension (SAH) and diabetes mellitus (DM)^5,6^. These conditions comprise a heterogeneous group of disorders associated with high morbidity and mortality and a significant socioeconomic impact, given their role as a leading cause of dialysis-dependent CKD in young adults^6,7,8,9^.

A 2018 international multicenter study analyzed 42,603 native kidney biopsies from 29 nephrology centers worldwide. In Latin America, lupus nephritis (LN) (38.1%) and focal segmental glomerulosclerosis (FSGS) (15.8%) were the most prevalent diagnoses^10^.

In 2010, a national survey on GD in Brazil included 9,617 renal biopsies, reporting that most patients were white (72.0%), female (51.0%), and from the Southeast region (47.0%), with a mean age of 35.07 ± 18.22 years. The primary indication for biopsy was nephrotic syndrome (NS) (39.0%), while acute kidney injury (AKI) was present in 14.4% of cases^11^. LN (45.5%) was the most common secondary glomerular disease (SGD), whereas FSGS (24.6%) was the most frequent primary glomerular disease (PGD)^11^, with a similar finding described in several previous Brazilian regional studies^12,13,14,15,16,17^.

The University Hospital of the Federal University of Juiz de Fora (HU/UFJF) provides nephrology care to patients from several municipalities in the Zona da Mata Mineira region. The hospital’s glomerular disease outpatient clinic serves as a reference center for the entire region. A previous study published in 2008 examined the epidemiological profile of GDs in the Zona da Mata Mineira region, analyzing 126 native kidney biopsies from adult patients between 1996 and 2006. The most common clinical presentation was NS (55.2%), while the most frequent histopathological diagnoses were FSGS (40.8%) among PGDs and LN (80.7%) among SGDs^18^.

The last evaluation of this population was conducted 19 years ago, revealing an epidemiological profile that appears to have changed. Thus, this study aimed to conduct a new analysis of GD patterns, identify predominant clinical and histopathological presentations, and assess factors associated with kidney disease progression and outcomes.

## Methods

A retrospective cohort study was conducted from January 1998 to January 2023. The sample consisted of adult patients seen at the GD outpatient clinic of the UFJF Nephrology Department.

The inclusion criteria were a confirmed diagnosis of GD (with or without kidney biopsy) and aged ≥18 years. The exclusion criteria were late-stage GD diagnosis (chronic glomerulonephritis), ongoing renal replacement therapy (RRT), or a previous history of kidney transplantation. Chronic glomerulonephritis was defined as severe loss of kidney function (eGFR < 20–30 mL/min per 1.73 m^2^) associated with extensive and irreversible renal damage (primarily interstitial fibrosis and tubular atrophy and/or bilateral renal atrophy), in which no ongoing therapeutic approach could reasonably alter the natural course of kidney function decline^7^.

Data were collected from patients’ medical records, with variables categorized based on admission, follow-up, and the end of follow-up. Sociodemographic, clinical, laboratory, renal outcome, and histopatholo­gical data were extracted.

The following variables were analyzed: admission variables (sociodemographic data, associated comorbidities, social and family history of nephropathy, initial nephrological presentation, and classification of GD as primary or secondary^7,8^); biopsy variables (age at biopsy, number of biopsies performed, diagnosis, and histopathological features).

SGD are defined as glomerular disorders with an identifiable underlying or systemic cause, such as infections, autoimmune, and rheumatic diseases (e.g., systemic lupus erythematosus and small vessel vasculitis), drug-induced glomerular injury (either direct or autoimmune-mediated), and cancers, including solid tumors and hematologic malignancies. In contrast, PGD are characterized by localized or intrinsic renal pathology^7,8,19^.

For FSGS, primary cases were defined as those presenting with full NS and no identifiable secondary cause. When available, electron microscopy confirmed these cases by demonstrating diffuse foot process effacement in podocytes. Secondary FSGS was characterized by the same histological pattern but was linked to underlying pathophysiological mechanisms, such as viral infections, drug exposure, glomerular hyperfiltration-induced injury, or adaptive processes. These cases typically lacked severe proteinuria and/or full NS, and electron microscopy, when available, did not reveal diffuse podocyte effacement^7,19^.

The outcome variables analyzed included initial and final eGFR, initial and final proteinuria, and clinical outcomes (active follow-up at the outpatient clinic, loss to follow-up, discharge to a CKD outpatient clinic, discharge from nephrology, initiation of RRT, and death).

Follow-up variables included follow-up duration, clinical data from initial and final consultations, body mass index (BMI), systolic (SBP) and diastolic blood pressure (DBP), and laboratory results at baseline and end of follow-up. Laboratory assessments included eGFR calculation using the Chronic Kidney Disease Epidemiology Collaboration (CKD-EPI) formula^20^ and proteinuria quantification through 24-hour urine collection or the protein-to-creatinine ratio from an isolated sample.

For patients who did not undergo kidney biopsy, GD diagnosis was based on persistent compatible clinical and laboratory findings, including dysmorphic hematuria and/or proteinuria, and the absence of diabetes mellitus, nephrolithiasis, or urinary tract infection^7,8,19^.

This study was conducted in accordance with the Declaration of Helsinki and was approved by the Ethics and Research Committee of the University Hospital of UFJF (CAAE: 47019521.9.0000.5133).

### Statistical Analysis

Descriptive statistical analysis results were reported as means, standard deviations, medians, or frequencies, depending on the type of variable (numerical or categorical) and its distribution (assessed using the Kolmogorov-Smirnov test). Comparative ana­lyses were conducted between the most frequent histopathological diagnoses and cases without renal biopsy. Statistical tests included the chi-square test, Student’s t-test, ANOVA, or the Mann-Whitney test, depending on the variable’s characteristics. Paired t-tests were conducted to compare initial versus final eGFR, while non-parametric tests were used for initial versus final proteinuria in related samples. Renal survival analysis was performed using Kaplan-Meier survival curves, with comparisons assessed using the log-rank test. Cox regression analysis was performed to evaluate renal survival as the outcome variable, adjusting for confounding factors with biological and/or statistical plausibility. The temporal evolution of histopathological diagnoses was analyzed, with frequencies expressed as percentages over specific time intervals. All statistical tests were conducted with a 95% confidence interval (CI). Statistical analyses were performed using SPSS version 17.0 (Chicago, Illinois^®^).

## Results

### Study Population

**Figure 1:**
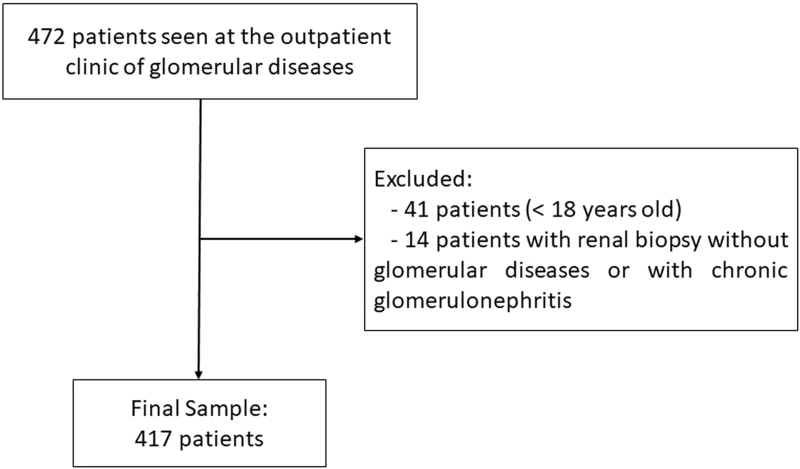
Study flowchart.

A total of 472 patients from the Nephrology GD outpatient clinic at UFJF were selected (January 1998 to January 2023). Forty-one patients were excluded as they were under the age of 18 and 14 were excluded as kidney biopsies indicated non-GD or chronic glomerulonephritis, leading to a final sample of 417 patients (Figure 1).

### Population Characteristics

Among the 417 patients, the mean age was 41.7 ± 14.4 years, with 57.3% were women and 69.8% reported being white. Most (66.7%) reported a family income of up to two minimum wages, 58.3% had an education level ranging from incomplete secondary education to higher education, and 72.1% were employed. A total of 323 patients (77.5%) underwent kidney biopsy, and 13% required a repeat biopsy for therapeutic or prognostic purposes.

**Figure 2:**
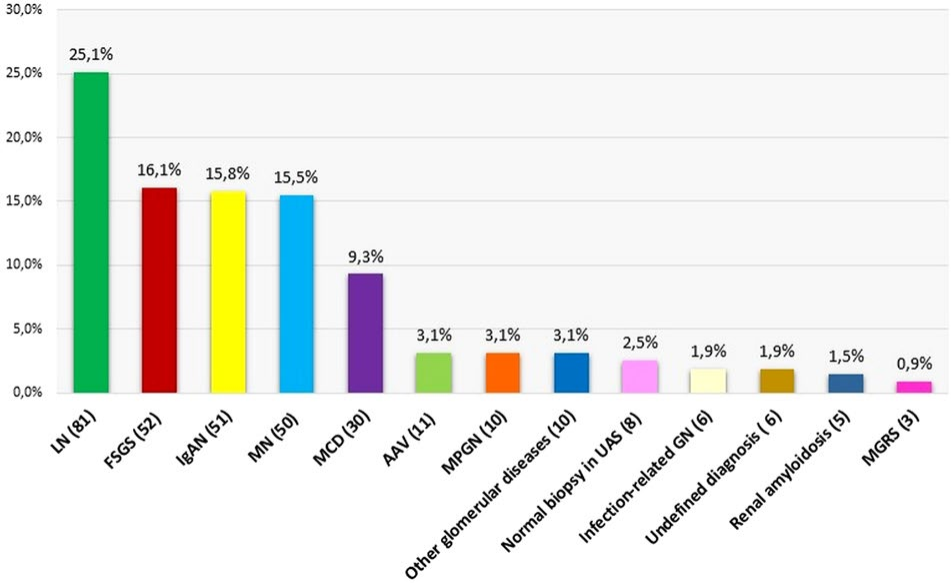
Histopathological diagnosis of glomerular diseases.

**Figure 3:**
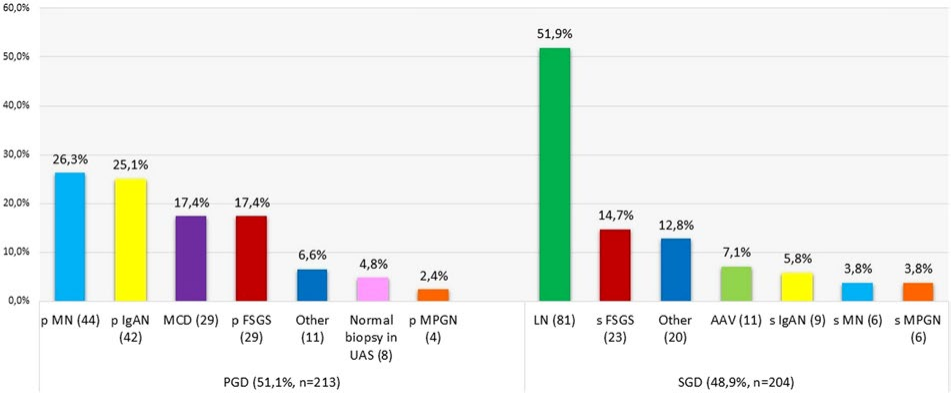
Histopathological diagnosis of primary and secondary glomerular diseases.

The most frequent histopathological diagnoses were LN, followed by FSGS, IgA nephropathy (IgAN), and membranous nephropathy (MN) (Figure 2). PGDs accounted for 51.1% of cases, with primary MN being the most frequent subtype. Among SGDs, LN and secondary FSGS were the most common conditions (Figure 3).

Abbreviations – LN (Lupus Nephritis), IgAN (IgA Nephropathy), MN (Membranous Nephropathy), FSGS (Focal and Segmental Glomerulosclerosis), MCD (Minimal Change Disease), Normal biopsy in UAS (Normal kidney biopsy in urinary abnormalities syndrome), MPGN (Membranoproliferative Glomerulonephritis), AAV (ANCA-associated vasculitis), Other glomerular diseases (fibrillary glomerulonephritis; non-IgA mesangial glomeru­lonephritis; crescentic glomerulonephritis not classified in previous GDs; thrombotic microangiopathy), Infection-related GN (Infection-related Glomerulonephritis), Undefined diagnosis (Undefined histological diagnosis), MGRS (Monoclonal gammopathy of renal significance).

Abbreviations – PGD (primary glomerular diseases), SGD (secondary glomerular diseases), LN (Lupus Nephritis), IgAN (IgA Nephropathy), MN (Membranous Nephropathy), FSGS (Focal and Segmental Glomerulosclerosis), MCD (Minimal Change Disease), Normal biopsy in UAS (Normal kidney biopsy in urinary abnormalities syndrome), MPGN (Membranoproliferative Glomerulonephritis), AAV (ANCA-associated vasculitis), Other (Other GD), p (primary), s (secondary).

The most common comorbidities were SAH, dyslipidemia, and psychiatric illnesses. Additionally, 29.7% of patients were current or former smokers, and 18.7% had CVD (Table 1). At baseline, 78.0% had a BMI ≥ 25 (Table 1).

**Table 1:** Total clinical data, by histological type of glomerular disease and in non-biopsied cases

Variable	Total	LN	IgAN	MN	FSGS	MCD	AAV	MPGN	Other^‡^	Non-biopsied	p
Age* (Years, Mean, SD)	41.7 (14.4)	34.8 (10.6)	37.5 (12.2)	48.9 (14.7)	40 (15)	38.3 (13.2)	48.7 (14.9)	52.3 (17.4)	47.0 (14.4)	43.9 (14.3)	**< 0.001**
White People (%)	69.8	49.4	82.0	78.0	79.6	55.2	100	80	75.7	69.1	**0.012**
Women (%)	57.3	82.7	47.1	36.0	40.4	50.0	26.4	50.0	57.9	67.0	**< 0.001**
Hypertension (%)	78.2	81.5	92.2	90.0	88.5	63.3	63.6	100.0	65.8	64.9	**< 0.001**
Diabetes (%)	17.3	16.0	11.8	28.0	19.2	20.0	27.3	0.0	26.3	10.6	0.098
Dyslipidemia (%)	47.2	48.1	49.0	62.0	55.8	53.3	54.5	50.0	34.2	35.1	0.062
Cardiovascular diseases (%)	18.7	11.1	9.8	26.0	32.7	20.0	18.2	40.0	15.8	16.0	**0.021**
Current or former smoking (%)	29.7	9.9	31.4	46.0	32.7	30	54.6	70	39.5	24.5	**< 0.001**
Psychiatric disease (%)	39.6	49.4	33.3	16.0	36.5	56.7	36.4	50.0	31.6	45.7	**0.004**
Bone disease (%)	16.1	17.3	19.6	20.0	9.6	6.7	18.2	40.0	13.2	16.0	0.320
SBP initial* (mmHg, Mean, SD)	135.3 (23.7)	139.6 (26.5)	137.3 (21.9)	135.2 (27.2)	139.1 (21.9)	125.1 (12.7)	123.1 (11.9)	165.6 (28.9)	134.4 (22.9)	130.1 (20.7)	**< 0.001**
DBP initial* (mmHg, Mean, SD)	85.1 (14.5)	87.7 (16.8)	87.4 (13.0)	83.8 (13.9)	87.9 (13.7)	80.4 (10.1)	77.8 (10.4)	97.4 (14.0)	83.7 (13.4)	82.3 (14.5)	**0.004**
BMI initial* (% ≥25, kg/m^2^)	78.0	71.6	82.4	70	76.9	80	81.9	80	76.4	85.1	0.600
Antiproteinuric (%)	89.2	93.8	98.0	96.0	100.0	100.0	90.9	80.0	81.6	71.3	**< 0.001**
SGLT2i (%)	8.6	0.0	31.4	4.0	13.5	3.3	18.2	10.0	5.3	5.3	**< 0.001**
Diuretics (%)	73.4	77.8	46.0	92.0	84.6	93.3	45.5	100	59.0	52.1	**< 0.001**
Statins (%)	64.0	53.1	60.8	88.0	78.8	80.0	90.9	80.0	57.9	46.8	**< 0.001**
Imunossupression (%)	61.1	100.0	54.9	72.9	55.8	93.3	100.0	50.0	26.3	28.0	**< 0.001**
Corticosteroids (%)	59.0	100.0	54.9	60.0	55.8	93.3	100.0	50.0	23.7	26.6	**< 0.001**
IV Pulse therapy (%)	30.7	80.2	23.5	36.0	11.5	10.0	81.8	40.0	10.5	7.4	**< 0.001**
Nephrologic presentation (%)											**< 0.001**
Nephrotic syndrome	33.8	22.2	9.8	92.0	53.8	96.7	0.0	10.0	15.8	8.5	
Nephritic syndrome	3.6	6.2	5.9	0.0	1.9	0.0	18.2	0.0	5.3	2.1	
Nephritic-nephrotic syndrome	10.1	24.7	11.8	0.0	0.0	0.0	45.5	60.0	7.9	2.1	
Isolated hematuria	7.2	0.0	2.0	0.0	0.0	0.0	9.1	0.0	7.9	26.6	
Isolated subnephrotic proteinuria	14.9	12.3	9.8	4.0	25.0	0.0	0.0	0.0	23.7	24.5	
Hematuria with subnephrotic proteinuria	24.2	17.3	58.8	2.0	13.5	0.0	18.2	30.0	34.2	33.0	
Isolated nephrotic proteinuria	4.8	17.3	0.0	2.0	1.9	3.3	9.1	0.0	5.3	0.0	
No data	1.4	0.0	2.0	0.0	3.8	0.0	0.0	0.0	0.0	3.2	
Initial AKI (%)	15.5	21.5	21.6	8.0	11.5	10.0	70.0	30.0	21.1	5.3	**< 0.001**
RPGN (%)	8.7	20.3	9.8	0.0	3.8	0.0	60.0	10.0	13.2	1.1	**< 0.001**
Initial dialysis (%)	4.3	7.6	2.0	0.0	5.8	0.0	30.0	10.0	10.5	0.0	**< 0.001**
Family history present (%)	23.3	9.9	33.3	16.0	26.9	10.0	9.1	0.0	34.2	35.1	**< 0.001**

Abbreviations – LN (Lupus Nephritis); IgAN (IgA Nephropathy); MN (Membranous Nephropathy); FSGS (Focal and Segmental Glomerulosclerosis; MCD (Minimal Change Disease); MPGN (Membranoproliferative Glomerulonephritis); AAV (ANCA-associated vasculitis); Other (Other glomerular disease: renal amyloidosis, monoclonal gammopathy of renal significance, glomerulonephritis associated with infection, normal biopsy with hematuria and/or subnephrotic proteinuria, fibrillary glomerulonephritis, non-IgA mesangial glomerulonephritis, crescentic glomerulonephritis not classified in previous glomerular diseases, thrombotic microangiopathy, no defined histological diagnosis); Non-biopsied (No kidney biopsy); SD (standard deviation); % (percentage); < (minor); ≥ (greater than or equal to); Cardiovascular diseases (Heart failure, or Coronary artery disease, or Cerebrovascular disease, or Peripheral arterial disease, or Rheumatic heart disease, excluding systemic arterial hypertension); SBP (systolic blood pressure); DBP (diastolic blood pressure); BMI (body mass index); Antiproteinuric (Angiotensin-converting enzyme inhibitors, or angiotensin II receptor blockers, or aldosterone blockers, or sodium-glucose cotransporter 2 inhibitors); SGLT2i (sodium-glucose cotransporter 2 inhibitors); Diuretics (loop diuretic, or thiazide diuretic, or aldosterone blocker); IV Pulse therapy (Intravenous Pulse Therapy); AKI (acute kidney injury); RPGN (rapidly progressive glomerulonephritis);

*(at the beginning of the follow-up).

Regarding medication use, 89.2% of patients were prescribed antiproteinuric drugs, 73.4% used diuretics, and 61.1% received immunosuppressive therapy, predominantly corticosteroids (59%). Pulse therapy with corticosteroids and/or cyclophosphamide was administered to 30.7% (Table 1).

The main nephrological clinical presentation was NS (33.8%), followed by hematuria with subnephrotic proteinuria (24.2%), and isolated subnephrotic proteinuria (14.9%). AKI was observed in 15.5% of patients, rapidly progressive glomerulonephritis (RPGN) in 8.7%, and 4.3% required dialysis (Table 1). Family history of nephropathy was reported in 23.3% (Table 1), with a higher prevalence among those with family members who required dialysis or kidney transplantation (37.4%).

Among patients who underwent kidney biopsy, 61.8% had interstitial fibrosis and tubular atrophy (IFTA), averaging 20.0% (±15.8%) of IFTA. Glomerular sclerosis was detected in 69.3%, vascular lesions in 44.4%, and crescents in 23.8% (predominantly cellular crescents, 40.5%) (Table 2).

**Table 2:** Histopathological, laboratory and clinical outcome data by histological type of glomerular disease and in non-biopsied cases

Variable	Total	LN	IgAN	MN	FSGS	MCD	AAV	MPGN	Other^‡^	Non-biopsied	p
eGFR (Mean, SD)											
Initial	72.9 (33.3)	79.7 (35.3)	60.0 (29.1)	78.1 (30.8)	67.8 (32.5)	91.8 (28.0)	54.5 (39.3)	42.6 (26.3)	65.3 (38.0)	76.3 (29.2)	**< 0.001**
Final	62.5 (33.0)	73.6 (34.8)	48.3 (28.0)	60.0 (32.8)	49.8 (28.5)	85.4 (24.7)	47.0 (32.5)	32.3 (15.7)	51.7 (31.7)	70.8 (30.8)	**< 0.001**
eGFR initial *vs*. final	**p < 0.001**										
Proteinuria (Mean, SD)											
Initial	3.7 (4.0)	2.9 (2.3)	2.9 (3.2)	7.9 (4.9)	5.0 (4.7)	7.6 (4.6)	2.1 (1.9)	3.3 (3.0)	2.6 (2.6)	1.3 (1.7)	**< 0.001**
Final	1.3 (2.1)	0.9 (1.9)	1.1 (1.3)	2.3 (2.9)	2.2 (2.2)	0.7 (1.5)	1.3 (1.3)	3.2 (5.2)	1.0 (1.0)	0.9 (2.0)	**< 0.001**
Proteinuria initial *vs*. final	**p < 0.001**										
Number of biopsies (%)											**< 0.001**
1	87.0	75.3	94.1	94.0	92.3	83.3	81.8	90.0	89.5	–	
≥2	13	24.7	5.9	6	7.7	16.7	18.2	10	10.5	–	
Crescents (%)	23.8	45.5	21.1	0.0	0.0	0.0	100.0	20.0	16.7	–	**< 0.001**
% of IFTA (mean, SD)	20.0 (15.8)	15.0 (12.5)	20.0 (19.1)	20.0 (13.2)	20.0 (15.8)	5.0 (5.0)	30.0 (16.8)	17.5 (12.2)	37.5 (18.5)	–	0.25
Total or partial glomerular sclerosis (%)	69.3	54.9	88.1	51.4	100	46.7	85.7	71.4	54.5	–	**< 0.001**
Vascular injury (%)	44.4	28.6	50.0	47.7	63.6	21.1	62.5	37.5	44.4	–	**0.010**
Follow-up in months (median, IQR)	49.0 (12–99)	52.0 (19–66)	64.0 (13–124)	68.0 (24.3–134.5)	50.0 (18.8–99)	77.5 (24.8–124)	22.0 (4.5–57.5)	16.5 (12.3–29.5)	19.0 (7.5–77.5)	40.0 (10–80)	**0.019**
Clinical outcome (%)											**0.002**
Active in outpatient clinic	61.4	77.8	62.7	62.0	59.6	63.3	72.7	50.0	44.7	53.2	
Loss to follow-up	22.3	13.6	17.6	22.0	15.4	33.3	9.1	10.0	31.6	31.9	
Transfer to conservative CKD outpatient clinic	9.8	4.9	15.7	10.0	11.5	3.3	9.1	10.0	13.2	10.6	
Dialysis	4.6	1.2	3.9	4.0	11.5	0.0	9.1	30.0	10.5	0.0	
Death	1.2	2.5	0.0	2.0	1.9	0.0	0.0	0.0	0.0	1.1	
Discharge from Nephrology follow-up	0.7	0.0	0.0	0.0	0.0	0.0	0.0	0.0	0.0	3.2	

Abbreviations – LN (Lupus Nephritis); IgAN (IgA Nephropathy); MN (Membranous Nephropathy); FSGS (Focal and Segmental Glomerulosclerosis; MCD (Minimal Change Disease); MPGN (Membranoproliferative Glomerulonephritis); AAV (ANCA-associated vasculitis); Other (Other glomerular disease: renal amyloidosis, monoclonal gammopathy of renal significance, glomerulonephritis associated with infection, normal biopsy with hematuria and/or subnephrotic proteinuria, fibrillary glomerulonephritis, non-IgA mesangial glomerulonephritis, crescentic glomerulonephritis not classified in previous glomerular diseases, thrombotic microangiopathy, no defined histological diagnosis); Non-biopsied (No kidney biopsy); eGFR (estimated glomerular filtration rate); SD(standard deviation); % (percentage); IQR (interquartile range); < (minor); = (greater than or equal to); IFTA (interstitial fibrosis and tubular atrophy); CKD (chronic kidney disease).

### Comparison of Histological Diagnoses and Non-Biopsied Cases

Patients with LN had the lowest average age, while those with membranoproliferative glomerulone­phritis (MPGN) had the highest age. LN was more frequent in females, whereas ANCA-associated vasculitis (AAV) and MN predominated in males. All cases of AAV were observed in white patients (Table 1).

MPGN was associated with 100% prevalence of SAH, whereas minimal change disease (MCD) had the lowest (63.3%). Psychiatric disorders were more common in patients with MCD, and MPGN patients had the highest prevalence of smoking. CVDs were more frequent in MPGN and FSGS patients (Table 1). MPGN was associated with significantly higher initial SBP (p < 0.001) and DBP (p = 0.004) (Table 1).

Medication usage varied across groups: antiproteinuric drugs were used by all MCD and FSGS patients and 71.3% of non-biopsied cases (p < 0.001). Diuretics were most frequently prescribed for MPGN (100%), MCD (93.3%), MN (92%), and FSGS (84.6%) patients (p < 0.001). Statins were most frequently prescribed in AAV (90.9%) and MN (88%) (p < 0.001), while SGLT2 inhibitors were mostly commonly used in IgAN patients (31.4%) (p < 0.001) (Table 1).

Immunosuppressive therapy was administered in all LN and AAV cases and was also prevalent in MCD (93.3%) and MN (72.9%) (p < 0.001). NS predominated in MCD (96.7%), MN (92%), and FSGS (53.8%) patients. RPGN was most frequent in AAV (60.0%) and LN (20.3%), whereas no cases were observed in MCD or MN (p < 0.001). AAV had the highest dialysis requirement (30.0%) (p < 0.001) (Table 1).

Histopathological analysis revealed more crescents in AAV (100%) and LN (45.5%) (p < 0.001), while IFTA was more common in MPGN (88.9%), IgAN (80%), and FSGS (79.1%) (p < 0.001). Glomerular sclerosis (total or segmental, excluding FSGS) was prevalent in IgAN (88.1%) and AAV (85.7%) cases but less frequent in MCD cases (46.7%) (p < 0.001) (Table 2).

### Outcomes

**Figure 4:**
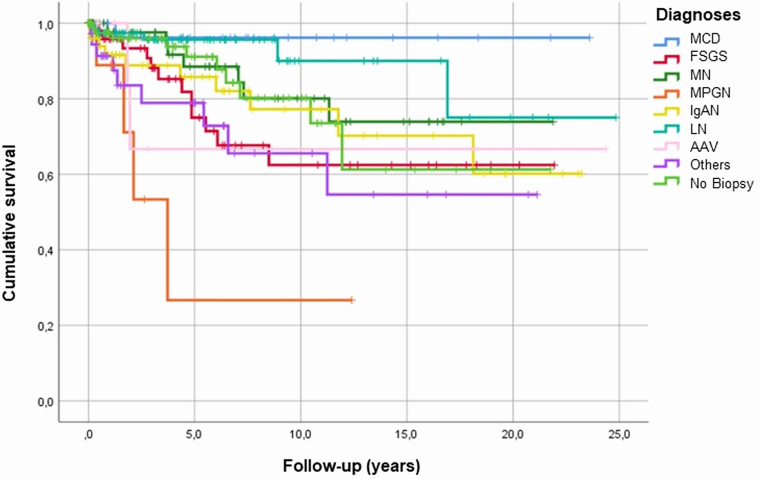
Renal survival curve (Kaplan-Meier) in the comparison of the main diagnoses of glomerular diseases (log rank 0.001).

During follow-up, the mean eGFR declined from 72.9 ± 33.3 to 62.5 ± 33.0 (p < 0.001), while proteinuria decreased from 3.7 ± 4.0 to 1.3 ± 2.1 (p < 0.001) (Table 2). MCD showed the best renal survival, while MPGN had the poorest outcomes (Figure 4). Most patients (61.4%) remained in follow-up, with low rates of chronic RRT (4.6%) or mortality (1.2%) (Table 2).

Abbreviations – LN (Lupus Nephritis), IgAN (IgA Nephropathy), MN (Membranous Nephropathy), FSGS (Focal and Segmental Glomerulosclerosis), MCD (Minimal Change Disease), MPGN (Membranoproliferative Glomerulonephritis), AAV (ANCA-associated vasculitis), Others (Others GD: renal amyloidosis; monoclonal gammopathy of renal significance; glomerulonephritis associated with infection; normal biopsy with hematuria and/or subnephrotic proteinuria; fibrillary glomerulonephritis; non-IgA mesangial glomerulonephritis; crescentic glomerulonephritis not classified in previous GDs; thrombotic microangiopathy; no defined histological diagnosis), No kidney biopsy (No Biopsy).

Cox regression analysis identified protective factors to CKD progression, including higher baseline eGFR (HR = 0.956, 95%CI: 0.920–0.994; p = 0.023). Risk factors for CKD progression included the presence of IFTA (HR = 1.079, 95%CI: 1.020–1.142; p = 0.008) and higher BMI (HR = 1.257, 95%CI: 1.016–1.556; p = 0.035) (Table 3).

**Table 3:** Cox regression analysis

Cox regression analysis: outcome renal survival adjusted for confounders			
	HR	95% CI	*p*
Age at diagnosis of GD	1.041	0.955–1.134	0.366
Sex feminine	0.163	0.022–1.211	0.076
Initial eGFR	0.956	0.920–0.994	**0.023**
Initial proteinuria	0.932	0.687–1.263	0.648
DM (diagnosis present)	0.985	0.153–6.348	0.988
Initial BMI	1.257	1.016–1.556	**0.035**
IFTA	1.079	1.020–1.142	**0.008**
ACE inhibitors or angiotensin receptor blocker (use)	83.3	0.420–16502.754	0.101
Immunosuppression (use)	0.626	0.080–4.881	0.655

Abbreviations – GD: glomerular disease; eGFR: estimated glomerular filtration rate; DM: diabetes mellitus; BMI: body mass index; IFTA: interstitial fibrosis and tubular atrophy; ACE: Angiotensin-converting enzyme inhibitor; IFTA: interstitial fibrosis and tubular atrophy; HR: Hazard ratio; CI: Confidence interval.

**Figure 5:**
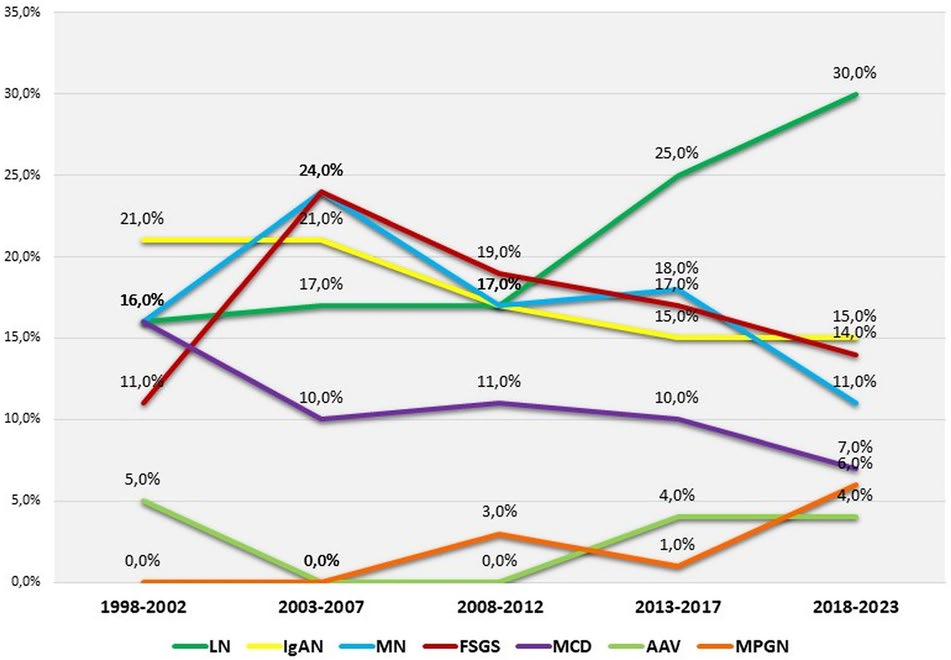
Temporal evolution of the prevalence of the main histopathological diagnoses of glomerular diseases from January 1998 to January 2023.

Over time, the prevalence of LN cases increased, while podocytopathies (MCD and FSGS) declined and IgAN remained stable (Figure 5).

Abbreviations – LN (Lupus Nephritis), IgAN (IgA Nephropathy), MN (Membranous Nephropathy), FSGS (Focal and Segmental Glomerulosclerosis), MCD (Minimal Change Disease), MPGN (Membranoproliferative Glomerulonephritis), AAV (ANCA-associated vasculitis).

## Discussion

Our study confirmed a shift in the epidemiological profile of GD in the region over the past 25 years. It provided new insights by incorporating renal outcome data alongside prevalence rates and histopathological diagnosis descriptions.

A multinational study examining GD frequency across Europe, North America, Latin America, and Asia reported a median age of 47.3 years (39.7–56.9), with 53% of patients being male and 57% identifying as white^10^. Latin American patients were younger and predominantly female, aligning with our findings. This demographic profile, characterized by a predominance of young female patients, is likely linked to the high prevalence of LN in Latin America and in Brazil, suggesting a possible genetic component^21^.

Regarding this aspect, a significant proportion of patients (23.3%) reported a family history of nephropathy, reinforcing the genetic influence on GD^22^. A genetic study of 163 patients with CKD of unknown etiology (2016–2020) identified a definitive genetic diagnosis in 31% of the 100 patients with GD^23^. Exome sequencing of 111 cases of idiopathic NS revealed no pathogenic variants in the corticosteroid-sensitive group, whereas 30% of corticosteroid-resistant cases harbored pathogenic variants in genes associated with podocytopathies^24^.

This population exhibited a high cardiovascular risk, with significant comorbidities and clinical events, aligning with previous studies that highlight the connection between traditional cardiovascular risk factors and GD-specific factors, including their treatment^25,26^. A cohort study of 1,912 patients with PGD (IgAN, FSGS, MCD, or MN) found that the cardiovascular event rate in this population was 2.5 times higher than in the general population, with FSGS patients exhibiting the highest risk^25^. A Danish population-based cohort of 1,644 systemic lupus erythematosus (SLE) patients identified a significantly higher risk of acute myocardial infarction (AMI) (eight-fold increase) and cardiovascular mortality (four-fold increase) in SLE patients with LN compared to those without LN^26^.

Additionally, psychiatric disorders were more frequent in disease subgroups requiring higher corticosteroid use—the primary immunosuppressive therapy in our study—reinforcing a well-established association^27^. A case-control study comparing 50 SLE patients with LN, 50 SLE patients without LN, and 50 healthy controls found a higher prevalence of anxiety and depression among LN patients, with increased risk in those of advanced age and with greater histological activity^28^. Another study investigating the association between psychiatric disorders and PGD involving 950 participants reported a 12% prevalence of psychiatric diagnoses. This diagnosis was significantly associated with CKD progression (HR = 2.45, 95% CI: 1.53–3.92)^29^.

Brazil is a country of continental size, and as such, regional variations in GD patterns are expected. Data from the 2017 Pernambuco Registry of Glomerulopathies the Northeast region reported that FSGS (43.0%) was the most common PGD, while LN (67.0%) was the predominant SGD^12^. A 2015 study in Ceará found nephrotic-range proteinuria in 67.3% of patients, with the most frequent histopathological diagnoses being FSGS (19.6%), MCD (17.9%), MN (16.7%), and LN (11.9%)^13^. Similarly, a 2016 survey from Southern Brazil found a higher prevalence of secondary glomerular diseases (61.2%), with LN (30.2%) being the most common diagnosis. Among PGDs, FSGS remained the most frequent (10.3%)^14^. A 2010 study from the Federal District reported FSGS (26.9%) as the most prevalent PGD, while LN (50.0%) was the leading SGD^15^. In Northern Brazil (Amazonas state), FSGS was also identified as the most common GD^16^. A five-year analysis of GD cases in São Paulo, published in the 2006 Paulista Registry of Glomerulopathies, was conducted with data from a multicenter study involving 11 centers that analyzed 1,844 renal biopsies. The most frequent PGDs were FSGS (29.7%), MN (20.7%), and IgAN (17.8%), while LN was the most common SGD (66.2%)^17^. The first report of the Brazilian Renal Biopsy Registry was recently published. The study evaluated 954 biopsies, representing all Brazilian regions. The main diagnosis of GD was LN (22.6%), followed by IgAN (13%) and FSGS (12.2%)^30^.

In our study, LN remained the most prevalent SGD in our region, corroborating findings from a 2018 international study and other Brazilian studies^10,11,12,13,14,15,16,17,18^. However, in the PGD analysis, FSGS was the third most common diagnosis, along with MCD, after MN and IgAN. This contrasts with previous studies that identified FSGS as the most frequent PGD^11,12,13,14,15,16,17,18^. This finding reflects advances in the understanding of GD pathophysiology, recognizing FSGS not as a single disease but as a histopathological pattern of glomerular injury associated with multiple patholo­gical mechanisms primarily targeting the podocyte^31^.

Regarding other histopathological findings, our analysis revealed a profile suggestive of advanced chronicity, highlighting the need for earlier kidney biopsy indications, even in cases of persistent glomerular hematuria and/or low-level proteinuria. A Japanese study involving 552,951 participants found hematuria in 26.5%, occasional proteinuria in 10.1%, and persistent proteinuria in 1.5%. Additionally, a decline in eGFR was associated with both isolated hematuria and its synergistic effect with proteinuria^32^.

Throughout follow-up, eGFR declined, while proteinuria levels improved. These findings likely reflect disease progression, with declining eGFR over time, and treatment effects—both immunosuppressive and non-immunosuppressive—on proteinuria evolution. Additional indicators of favorable outcomes include most patients remaining under active follow-up, with low rates of progression to RRT and/or mortality.

Renal survival was highest in MCD and lowest in MPGN. In our study, MCD patients were younger, had lower IFTA and vascular lesion scores, and exhibited higher initial and final eGFR. They also showed greater reductions in proteinuria, suggesting a positive therapeutic response and disease remission. A risk analysis study on CKD progression, RRT requirement, cardiovascular events, and mortality in 907 adults with primary NS (MCD, FSGS, or MN) compared to a healthy control group found lower risks in MCD patients, except for venous thromboembolism^33^. MPGN patients in our study had the highest mean age, histological indicators suggestive of chronicity, lower initial and final eGFR, and minimal improvement in proteinuria, indicating a poorer therapeutic response. A Brazilian study analyzing 53 MPGN cases reported partial or complete remission in only 39% of patients^34^.

Renal survival in non-biopsied cases was similar to that of IgAN. A recent study found worse outcomes in IgAN and FSGS, with higher rates of progression to RRT^35^. These findings further emphasize the need to reconsider kidney biopsy indications for persistent urinary abnormalities that may not traditionally warrant histopathological evaluation, as well as the importance of expanding genetic analyses^22,23^.

The primary factors associated with CKD progression were lower initial eGFR, presence of IFTA, and higher BMI. The association between lower baseline eGFR and worse outcomes may reflect the selection of cases with greater clinical and/or histopathological severity at disease onset. A Korean study analyzing 1,943 renal biopsies from PGD patients found worse disease progression in those with an eGFR below 60 mL/min/1.73m^2,36^. Additionally, IFTA severity is a finding classically associated with chronicity and poorer outcomes^37^. Obesity is a well-established risk factor for CKD and has been specifically linked to obesity-associated glomerulopathy^38^. A systematic review and meta-analysis of IgAN cases found that overweight and obese patients had lower eGFR than those with a normal BMI^39^. A Japanese study analyzing 489 LN patients identified BMI and baseline eGFR as independent risk factors for CKD progression^40^.

The main limitations of this study include the extended analysis period, which led to the loss of follow-up of some patients, and constraints in data collection due to its retrospective design, as it was restricted to medical record analysis. Similar to most studies on this topic, our analysis focused on histopathological patterns of glomerular lesions rather than their underlying pathophysiology^10,11,12,13,14,15,16,17,18^. The next logical step is to evaluate each lesion subgroup, including those not subjected to histological analysis. This additional analysis should incorporate descriptive, analytical, and inferential methods, integrating new clinical, laboratory, and genetic approaches.

In conclusion, LN remained the most prevalent SGD in our region, while FSGS and MCD ranked third among PGDs, after MN and IgAN. Renal survival was highest in MCD and poorest in MPGN. Understanding the epidemiological profile of GD in our environment is crucial for developing health policies aimed at comprehensive care for this specific group of kidney disease patients.

## Data Availability

Data will be shared upon reasonable request to the corresponding author.
