# Supplementary material for: Epidemiological profile and renal outcomes in a 25-year Brazilian cohort of glomerular diseases
Source: J Bras Nefrol. 2025 Aug 15;47(4):e20240213. doi: 10.1590/2175-8239-JBN-2024-0213en (PMC12356627; doi:10.1590/2175-8239-JBN-2024-0213en)
Supplement: Translation

# Translation: Perfil epidemiológico e desfechos renais em uma coorte Brasileira de 25 anos de doenças glomerulares

**DOI:** 10.1590/2175-8239-JBN-2024-0213pt

**Authors:** Fernando Sales, Priscylla Vieira do Carmo, Rosália Maria Nunes Henriques Huaira, Nicolas William Gonçalves de Almeida, Mateus Henrique Toledo Lourenço, Natália Maria da Silva Fernandes

**Affiliations:** 1 Universidade Federal de Juíz de Fora, Hospital Universitário, Juiz de Fora, MG, Brasil.

**Keywords:** Estudos de Coortes, Insuficiência Renal Crônica, Epidemiologia, Glomerulonefrite, Patologia

## Abstract

**Introdução:**

Doenças glomerulares (DG) constituem importante causa de doença renal crônica (DRC). Este estudo teve como objetivo analisar perfis sociodemográfico, clínico e de desfechos renais em pacientes com DG.

**Métodos:**

Realizou-se estudo de coorte retrospectivo entre 1998–2023, com pacientes com idade ≥ 18 anos, diagnosticados com DG e atendidos em um hospital universitário no Brasil. Foram analisadas variáveis sociodemográficas, clínicas, histopatológicas e renais. Realizada análise comparativa entre os diagnósticos histopatológicos mais frequentes. A variável de desfecho foi a sobrevida renal.

**Resultados:**

Foram avaliados 417 pacientes, 57,3% eram mulheres e 69,8% brancos. A idade média foi 41,7 ± 14,4 anos. Doenças glomerulares primárias (DGP) representaram 51,1%, e 77,5% dos pacientes foram submetidos à biópsia renal. A DGP mais frequente foi nefropatia membranosa (26,3%), enquanto, entre as doenças glomerulares secundárias (DGS), nefrite lúpica (NL) foi a mais comum (51,9%). Doença de lesão mínima associou-se a melhor sobrevida renal, enquanto glomerulonefrite membra­noproliferativa apresentou piores desfechos (p = 0,001). Com relação à progressão da DRC, taxa de filtração glomerular estimada (TFGe) inicial maior foi fator de proteção (HR = 0,956, IC95%: 0,920–0,994; p = 0,023), ao passo que a presença de fibrose intersticial/atrofia tubular (FIAT) (HR = 1,079, IC95%: 1,020–1,142; p = 0,008) e índice de massa corporal (IMC) mais alto (HR = 1,257, IC95%: 1,016–1,556; p = 0,035) foram associados a um risco aumentado de progressão da DRC.

**Conclusão:**

A NL manteve-se como a DGS mais comum em nossa região, enquanto a glomeruloesclerose segmentar e focal foi a terceira DGP mais comum. Fatores de risco para piores desfechos incluíram maior IMC, menor TFGe e maior FIAT.

## Introdução

A doença renal crônica (DRC) é considerada um dos maiores desafios de saúde pública em todo o mundo, além de ser um fator de risco independente para doenças cardiovasculares (DCV)^1,2^. A prevalência global estimada da DRC, referente aos cinco estágios da doença, é de 13,4% (11,7–15,1%) e afeta mais de 800 milhões de pessoas^3,4^. No Brasil, a prevalência nacional de pacientes em diálise crônica aumentou de 665 por milhão de habitantes em 2019 para 758 em 2022^5^.

As doenças glomerulares (DG) representam a terceira principal causa de DRC dialítica no Brasil e em diversos outros países, atrás apenas da hipertensão arterial sistêmica (HAS) e do diabetes mellitus (DM)^5,6^. Essas condições compreendem um grupo heterogêneo de distúrbios associados à elevada morbidade e mortalidade e a um impacto socioeconômico significativo, dado seu papel como a mais frequente causa de DRC dependente de diálise em adultos jovens^6–9^.

Um estudo multicêntrico internacional realizado em 2018 analisou 42.603 biópsias de rins nativos provenientes de 29 centros de nefrologia em todo o mundo. Na América Latina, os diagnósticos mais prevalentes foram a nefrite lúpica (NL) (38,1%) e a glomeruloesclerose segmentar e focal (GESF) (15,8%)^10^.

Em 2010, uma pesquisa nacional sobre DG no Brasil incluiu 9.617 biópsias renais e relatou que a maioria dos pacientes era branca (72,0%), do sexo feminino (51,0%) e oriunda da região Sudeste (47,0%), com idade média de 35,07 ± 18,22 anos. A principal indicação para biópsia foi a síndrome nefrótica (SN) (39,0%) e a injúria renal aguda (IRA) esteve presente em 14,4% dos casos^11^. A NL (45,5%) foi a doença glomerular secundária (DGS) mais comum, ao passo que a GESF (24,6%) foi a doença glomerular primária (DGP)^11^ mais frequente, com achado semelhante descrito em diversos estudos regionais brasileiros anteriores^12–17^.

O Hospital Universitário da Universidade Federal de Juiz de Fora (HU/UFJF) presta atendimento nefrológico a pacientes de diversos municípios da região da Zona da Mata Mineira. O ambulatório de DG do hospital atua como um centro de referência para toda a região. Um estudo anterior, publicado em 2008, examinou o perfil epidemiológico das DG na região da Zona da Mata Mineira, com análise de 126 biópsias de rins nativos de pacientes adultos entre 1996 e 2006. A apresentação clínica mais comum foi a SN (55,2%), enquanto os diagnósticos histopatológicos mais frequentes foram a GESF (40,8%) entre as DGP, e a NL (80,7%) entre as secundárias^18^.

A última avaliação dessa população foi realizada há 19 anos, a qual revelou um perfil epidemiológico que aparenta ter se modificado ao longo do tempo. Assim, o presente estudo teve como objetivo realizar uma nova análise dos padrões de DG, identificar as apresentações clínicas e histopatológicas predo­minantes, bem como avaliar os fatores associados à progressão e aos desfechos da doença renal.

## Métodos

Foi realizado um estudo de coorte retrospectivo de janeiro de 1998 a janeiro de 2023. A amostra foi composta por pacientes adultos atendidos no ambulatório de DG do Departamento de Nefrologia da UFJF. Os critérios de inclusão foram um diagnóstico confirmado de DG (com ou sem biópsia renal) e idade ≥18 anos. Os critérios de exclusão foram: diagnóstico de DG em estágio avançado (glomerulonefrite crônica), terapia renal substitutiva (TRS) em curso ou histórico de transplante renal. A glomerulonefrite crônica foi definida como uma perda grave da função renal (TFGe < 20–30 mL/min por 1,73 m^2^) associada a danos renais extensos e irreversíveis (principalmente fibrose intersticial e atrofia tubular avançadas e/ou atrofia renal bilateral), nos quais nenhuma abordagem terapêutica em andamento poderia razoavelmente alterar o curso natural do declínio da função renal^7^.

Os dados foram coletados dos registros médicos dos pacientes, com variáveis categorizadas de acordo com a admissão, o acompanhamento e o término do seguimento. Foram extraídos dados sociodemográficos, clínicos, laboratoriais, histopa­tológicos e de desfechos renais.

As seguintes variáveis foram analisadas: variáveis de admissão (dados sociodemográficos, comorbidades associadas, histórico social e familiar de nefropatia, apresentação nefrológica inicial e classificação da DG como primária ou secundária^7,8^) e variáveis de biópsia (idade na biópsia, número de biópsias realizadas, diagnóstico e características histopatológicas).

As DGS são definidas como distúrbios glomeru­lares com causa subjacente ou sistêmica identificável, como infecções, doenças autoimunes e reumáticas (por exemplo, lúpus eritematoso sistêmico e vasculites de pequenos vasos), lesões glomerulares induzidas por medicamentos (sejam diretas ou mediadas por autoimunidade) e neoplasias, como tumores sólidos e malignidades hematológicas. Em contraste, as DGP são caracterizadas por patologia renal localizada ou intrínseca^7,8,19^.

Para a GESF, os casos primários foram definidos como aqueles que apresentaram SN completa e nenhuma causa secundária identificável. Quando disponível, a microscopia eletrônica confirmou esses casos ao demonstrar o apagamento difuso dos processos podais nos podócitos. A GESF secundária foi caracterizada pelo mesmo padrão histológico, mas foi associada a mecanismos fisiopatológicos subjacentes, como infecções virais, exposição a medicamentos, lesão induzida por hiperfiltração glomerular ou processos adaptativos. Esses casos normalmente não apresentaram proteinúria grave e/ou SN completa, e a microscopia eletrônica, quando disponível, não revelou apagamento difuso de podócitos^7,19^.

As variáveis de desfecho analisadas incluíram TFGe inicial e final, proteinúria inicial e final e desfechos clínicos (acompanhamento ambulatorial ativo, perda de seguimento, alta para um ambulatório de DRC, alta da nefrologia, início de TRS e óbito).

As variáveis de acompanhamento incluíram a duração do seguimento, dados clínicos das consultas inicial e final, índice de massa corporal (IMC), pressão arterial sistólica (PAS) e diastólica (PAD) e resultados laboratoriais no início e no final do acompanhamento. As avaliações laboratoriais compreenderam o cálculo da TFGe utilizando a fórmula *Chronic Kidney Disease Epidemiology Collaboration* (CKD-EPI)^20^ e quantificação da proteinúria por meio da coleta de urina de 24 horas ou da relação proteína/creatinina de uma amostra isolada.

Para os pacientes que não foram submetidos à biópsia renal, o diagnóstico de DG foi estabelecido com base em achados clínicos e laboratoriais compatíveis persistentes, tais como hematúria dismórfica e/ou proteinúria, e na ausência de diabetes mellitus, nefrolitíase ou infecção do trato urinário^7,8,19^.

Este estudo foi realizado em conformidade com a Declaração de Helsinque e foi aprovado pelo Comitê de Ética e Pesquisa do Hospital Universitário da UFJF (CAAE: 47019521.9.0000.5133).

### Análise Estatística

Os resultados da análise estatística descritiva foram relatados como médias, desvios-padrão, medianas ou frequências, a depender do tipo de variável (numérica ou categórica) e de sua distribuição (avaliada pelo teste de Kolmogorov-Smirnov). Foram realizadas análises comparativas entre os diagnósticos histopatológicos mais frequentes e os casos sem biópsia renal. Os testes estatísticos incluíram o teste qui-quadrado, o teste t de Student, ANOVA ou o teste de Mann-Whitney, conforme a característica da variável. Testes t pareados foram realizados para comparar a TFGe inicial e final, enquanto testes não paramétricos foram utilizados para a comparação da proteinúria inicial e final em amostras relacionadas. A análise de sobrevida renal foi realizada por meio de curvas de sobrevida de Kaplan-Meier, com comparações avaliadas pelo teste de log-rank. A análise de regressão de Cox foi empregada para avaliar a sobrevida renal como variável de desfecho, com ajuste para fatores de confusão com plausibilidade biológica e/ou estatística. A evolução temporal dos diagnósticos histopatológicos foi analisada, com as frequências expressas como porcentagens ao longo de intervalos de tempo específicos. Todos os testes estatísticos foram realizados com um intervalo de confiança (IC) de 95%. As análises estatísticas foram conduzidas com uso do programa SPSS, versão 17.0 (Chicago, Illinois^®^).

## Resultados

### População do Estudo

Foram selecionados 472 pacientes do ambulatório de Nefrologia especializado em DG da UFJF (janeiro de 1998 a janeiro de 2023). Quarenta e um pacientes foram excluídos por terem menos de 18 anos de idade e 14 foram excluídos pelo fato de as biópsias renais indicarem glomerulonefrite crônica ou não-DG, o que resultou em uma amostra final de 417 pacientes (Figura 1).

**Figura 1:** Fluxograma do estudo.

### Características da População

Entre os 417 pacientes, a média de idade foi de 41,7 ± 14,4 anos, 57,3% eram do sexo feminino e 69,8% autodeclarados brancos. A maioria (66,7%) relatou uma renda familiar de até dois salários mínimos, 58,3% possuíam um nível de escolaridade que variou do ensino médio incompleto ao ensino superior e 72,1% estavam empregados. Um total de 323 pacientes (77,5%) foram submetidos à biópsia renal e em 13% houve necessidade de repetir a biópsia para fins terapêuticos ou prognósticos.

O diagnóstico histopatológico mais frequente foi de NL, seguido por GESF, nefropatia por IgA (NIgA) e nefropatia membranosa (NM) (Figura 2). As DGP foram responsáveis por 51,1% dos casos e a NM primária o subtipo mais frequente. Entre as DGS, a NL e a GESF secundária foram as condições mais comuns (Figura 3).

**Figura 2:** Diagnóstico histopatológico das doenças glomerulares.

Abreviações – NL (Nefrite Lúpica), NIgA (Nefropatia por IgA), NM (Nefropatia Membranosa), GESF (Glomeruloesclerose Segmentar e Focal), DLM (Doença de Lesão Mínima), Biópsia normal na SAU (Biópsia renal normal na síndrome de anormalidades urinárias), GNMP (Glomerulonefrite Membranoproliferativa), ANCAV (Vasculite associada a ANCA), Outras doenças glomerulares (glomerulonefrite fibrilar; glomerulonefrite mesangial não IgA; glomerulonefrite crescêntica não classificada nas DG anteriores; microangiopatia trombótica), GN relacionada à infecção (glomerulonefrite relacionada à infecção), diagnóstico indefinido (diagnóstico histológico indefinido), GMSR (gamopatia monoclonal de significado renal).

**Figura 3:** Diagnóstico histopatológico das doenças glomerulares primárias e secundárias.

Abreviações – DGP (doenças glomerulares primárias), DGS (doenças glomerulares secundárias), NL (Nefrite lúpica), NIgA (Nefropatia por IgA), NM (Nefropatia Membranosa), GESF (Glomeruloesclerose Segmentar e Focal), DLM (Doença de Lesão Mínima), Biópsia normal na SAU (biópsia renal normal na síndrome de anormalidades urinárias), GNMP (Glomerulonefrite membranoproliferativa), ANCAV (Vasculite associada a ANCA), Outras (Outras DG), p (primária), s (secundária).

As comorbidades mais comuns foram HAS, dislipidemia e doenças psiquiátricas. Além disso, 29,7% dos pacientes eram fumantes ou ex-fumantes e 18,7% apresentaram DCV (Tabela 1). Na avaliação inicial, 78,0% tinham um IMC ≥ 25 (Tabela 1).

**Tabela 1:** Dados clínicos totais, por tipo histológico de doença glomerular e em casos não biopsiados

Variável	Total	NL	NIgA	NM	GESF	DLM	ANCAV	GNMP	Outras^‡^	Não biopsiados	p
Idade* (Anos, Média, DP)	41,7 (14,4)	34,8 (10,6)	37,5 (12,2)	48,9 (14,7)	40 (15)	38,3 (13,2)	48,7 (14,9)	52,3 (17,4)	47,0 (14,4)	43,9 (14,3)	**< 0,001**
Pessoas Brancas (%)	69,8	49,4	82,0	78,0	79,6	55,2	100	80	75,7	69,1	**0,012**
Mulheres (%)	57,3	82,7	47,1	36,0	40,4	50,0	26,4	50,0	57,9	67,0	**< 0,001**
Hipertensão (%)	78,2	81,5	92,2	90,0	88,5	63,3	63,6	100,0	65,8	64,9	**< 0,001**
Diabetes (%)	17,3	16,0	11,8	28,0	19,2	20,0	27,3	0,0	26,3	10,6	0,098
Dislipidemia (%)	47,2	48,1	49,0	62,0	55,8	53,3	54,5	50,0	34,2	35,1	0,062
Doenças cardiovasculares (%)	18,7	11,1	9,8	26,0	32,7	20,0	18,2	40,0	15,8	16,0	**0,021**
Tabagismo atual ou anterior (%)	29,7	9,9	31,4	46,0	32,7	30	54,6	70	39,5	24,5	**< 0,001**
Doença psiquiátrica (%)	39,6	49,4	33,3	16,0	36,5	56,7	36,4	50,0	31,6	45,7	**0,004**
Doença óssea (%)	16,1	17,3	19,6	20,0	9,6	6,7	18,2	40,0	13,2	16,0	0,320
PAS inicial* (mmHg, Média, DP)	135,3 (23,7)	139,6 (26,5)	137,3 (21,9)	135,2 (27,2)	139,1 (21,9)	125,1 (12,7)	123,1 (11,9)	165,6 (28,9)	134,4 (22,9)	130,1 (20,7)	**< 0,001**
PAD inicial* (mmHg, Méda, DP)	85,1 (14,5)	87,7 (16,8)	87,4 (13,0)	83,8 (13,9)	87,9 (13,7)	80,4 (10,1)	77,8 (10,4)	97,4 (14,0)	83,7 (13,4)	82,3 (14,5)	**0,004**
IMC inicial* (% ≥25, kg/m^2^)	78,0	71,6	82,4	70	76,9	80	81,9	80	76,4	85,1	0,600
Antiproteinúrico (%)	89,2	93,8	98,0	96,0	100,0	100,0	90,9	80,0	81,6	71,3	**< 0,001**
iSGLT2 (%)	8,6	0,0	31,4	4,0	13,5	3,3	18,2	10,0	5,3	5,3	**< 0,001**
Diuréticos (%)	73,4	77,8	46,0	92,0	84,6	93,3	45,5	100	59,0	52,1	**< 0,001**
Estatinas (%)	64,0	53,1	60,8	88,0	78,8	80,0	90,9	80,0	57,9	46,8	**< 0,001**
Imunossupressão (%)	61,1	100,0	54,9	72,9	55,8	93,3	100,0	50,0	26,3	28,0	**< 0,001**
Corticosteroides (%)	59,0	100,0	54,9	60,0	55,8	93,3	100,0	50,0	23,7	26,6	**< 0,001**
Pulsoterapia IV (%)	30,7	80,2	23,5	36,0	11,5	10,0	81,8	40,0	10,5	7,4	**< 0,001**
Apresentação nefrológica (%)											**< 0,001**
Síndrome nefrótica	33,8	22,2	9,8	92,0	53,8	96,7	0,0	10,0	15,8	8,5	
Síndrome nefrítica	3,6	6,2	5,9	0,0	1,9	0,0	18,2	0,0	5,3	2,1	
Síndrome nefrítica-nefrótica	10,1	24,7	11,8	0,0	0,0	0,0	45,5	60,0	7,9	2,1	
Hematúria isolada	7,2	0,0	2,0	0,0	0,0	0,0	9,1	0,0	7,9	26,6	
Proteinúria subnefrótica isolada	14,9	12,3	9,8	4,0	25,0	0,0	0,0	0,0	23,7	24,5	
Hematúria com proteinúria subnefrótica	24,2	17,3	58,8	2,0	13,5	0,0	18,2	30,0	34,2	33,0	
Proteinúria nefrótica isolada	4,8	17,3	0,0	2,0	1,9	3,3	9,1	0,0	5,3	0,0	
Sem dados	1,4	0,0	2,0	0,0	3,8	0,0	0,0	0,0	0,0	3,2	
IRA inicial (%)	15,5	21,5	21,6	8,0	11,5	10,0	70,0	30,0	21,1	5,3	**< 0,001**
GNRP (%)	8,7	20,3	9,8	0,0	3,8	0,0	60,0	10,0	13,2	1,1	**< 0,001**
Diálise inicial (%)	4,3	7,6	2,0	0,0	5,8	0,0	30,0	10,0	10,5	0,0	**< 0,001**
Histórico familiar presente (%)	23,3	9,9	33,3	16,0	26,9	10,0	9,1	0,0	34,2	35,1	**< 0,001**

Legenda: NL (Nefrite Lúpica); NIgA (Nefropatia por IgA); NM (Nefropatia Membranosa); GESF (Glomeruloesclerose Segmentar e Focal); DLM (Doença de Lesão Mínima); GNMP (Glomerulonefrite Membranoproliferativa); ANCAV (Vasculite associada a ANCA); Outras (Outras doenças glomerulares: amiloidose renal, gamopatia monoclonal de significado renal, glomerulonefrite associada à infecção, biópsia normal com hematúria e/ou proteinúria subnefrótica, glomerulonefrite fibrilar, glomerulonefrite mesangial não IgA, glomerulonefrite crescêntica não classificada nas doenças glomerulares anteriores, microangiopatia trombótica, sem diagnóstico histológico definido); Não biopsiados (sem biópsia renal); DP (desvio padrão); % (porcentagem); < (menor); ≥ (maior ou igual); Doenças cardiovasculares (insuficiência cardíaca, ou doença arterial coronariana, ou doença cerebrovascular, ou doença arterial periférica, ou cardiopatia reumática, excluindo hipertensão arterial sistêmica); PAS (pressão arterial sistólica); PAD (pressão arterial diastólica); IMC (índice de massa corporal); Antiproteinúricos (inibidores da enzima conversora de angiotensina, ou bloqueadores do receptor de angiotensina II, ou bloqueadores de aldosterona, ou inibidores do cotransportador de sódio-glicose tipo 2); iSGLT2 (inibidores do cotransportador de sódio-glicose tipo 2); Diuréticos (diurético de alça, diurético tiazídico ou bloqueador de aldosterona); Pulsoterapia IV (pulsoterapia intravenosa); IRA (injúria renal aguda); GNRP (glomerulonefrite rapidamente progressiva);

*(no início do acompanhamento).

Com relação ao uso de medicamentos, 89,2% dos pacientes receberam prescrição de antipro­teinúricos, 73,4% utilizaram diuréticos e 61,1% receberam terapia imunossupressora, predomi­nantemente corticosteroides (59%). A pulsoterapia com corticosteroides e/ou ciclofosfamida foi administrada a 30,7% (Tabela 1).

A principal apresentação clínica nefrológica foi a SN (33,8%), seguida por hematúria com proteinúria subnefrótica (24,2%) e proteinúria subnefrótica isolada (14,9%). A IRA foi observada em 15,5% dos pacientes, a glomerulonefrite rapidamente progressiva (GNRP) em 8,7% e 4,3% necessitaram de diálise (Tabela 1). O histórico familiar de nefropatia foi relatado em 23,3% (Tabela 1), com maior prevalência entre aqueles com familiares que necessitaram de diálise ou transplante renal (37,4%).

Entre os pacientes submetidos à biópsia renal, 61,8% apresentaram fibrose intersticial e atrofia tubular (FIAT), com média de 20,0% (± 15,8%) de acometimento. A esclerose glomerular foi detectada em 69,3%, as lesões vasculares em 44,4% e as crescentes em 23,8% (predominantemente crescentes celulares – 40,5%) (Tabela 2).

**Tabela 2:** Dados histopatológicos, laboratoriais e de desfechos clínicos por tipo histológico de doença glomerular e em casos não biopsiados

Variável	Total	NL	NIgA	NM	GESF	DLM	ANCAV	GNMP	Outras^‡^	Não biopsiados	p
TFGe (Média, DP)											
Inicial	72,9 (33,3)	79,7 (35,3)	60,0 (29,1)	78,1 (30,8)	67,8 (32,5)	91,8 (28,0)	54,5 (39,3)	42,6 (26,3)	65,3 (38,0)	76,3 (29,2)	**< 0,001**
Final	62,5 (33,0)	73,6 (34,8)	48,3 (28,0)	60,0 (32,8)	49,8 (28,5)	85,4 (24,7)	47,0 (32,5)	32,3 (15,7)	51,7 (31,7)	70,8 (30,8)	**< 0,001**
TFGe inicial *vs*. final	**p < 0,001**										
Proteinúria (Média, DP)											
Inicial	3,7 (4,0)	2,9 (2,3)	2,9 (3,2)	7,9 (4,9)	5,0 (4,7)	7,6 (4,6)	2,1 (1,9)	3,3 (3,0)	2,6 (2,6)	1,3 (1,7)	**< 0,001**
Final	1,3 (2,1)	0,9 (1,9)	1,1 (1,3)	2,3 (2,9)	2,2 (2,2)	0,7 (1,5)	1,3 (1,3)	3,2 (5,2)	1,0 (1,0)	0,9 (2,0)	**< 0,001**
Proteinúria inicial *vs*. final	**p < 0,001**										
Número de biópsias (%)											**< 0,001**
1	87,0	75,3	94,1	94,0	92,3	83,3	81,8	90,0	89,5	–	
≥2	13	24,7	5,9	6	7,7	16,7	18,2	10	10,5	–	
Crescentes (%)	23,8	45,5	21,1	0,0	0,0	0,0	100,0	20,0	16,7	–	**< 0,001**
% de FIAT (média, DP)	20,0 (15,8)	15,0 (12,5)	20,0 (19,1)	20,0 (13,2)	20,0 (15,8)	5,0 (5,0)	30,0 (16,8)	17,5 (12,2)	37,5 (18,5)	–	0,25
Esclerose glomerular total ou parcial (%)	69,3	54,9	88,1	51,4	100	46,7	85,7	71,4	54,5	–	**< 0,001**
Lesão vascular (%)	44,4	28,6	50,0	47,7	63,6	21,1	62,5	37,5	44,4	–	**0,010**
Acompanhamento em meses (mediana, IIQ)	49,0 (12-99)	52,0 (19-66)	64,0 (13-124)	68,0 (24,3-134,5)	50,0 (18,8-99)	77,5 (24,8-124)	22,0 (4,5-57,5)	16,5 (12,3-29,5)	19,0 (7,5-77,5)	40,0 (10-80)	**0,019**
Desfecho clínico (%)											**0,002**
Acompanhamento ambulatorial ativo	61,4	77,8	62,7	62,0	59,6	63,3	72,7	50,0	44,7	53,2	
Perda de acompanhamento	22,3	13,6	17,6	22,0	15,4	33,3	9,1	10,0	31,6	31,9	
Encaminhamento para DRC conservador	9,8	4,9	15,7	10,0	11,5	3,3	9,1	10,0	13,2	10,6	
Diálise	4,6	1,2	3,9	4,0	11,5	0,0	9,1	30,0	10,5	0,0	
Óbito	1,2	2,5	0,0	2,0	1,9	0,0	0,0	0,0	0,0	1,1	
Alta do acompanhamento nefrológico	0,7	0,0	0,0	0,0	0,0	0,0	0,0	0,0	0,0	3,2	

Legenda: NL (Nefrite Lúpica); NIgA (Nefropatia por IgA); NM (Nefropatia Membranosa); GESF (Glomeruloesclerose Segmentar e Focal); DLM (Doença de Lesão Mínima); GNMP (Glomerulonefrite Membranoproliferativa); ANCAV (Vasculite associada a ANCA); Outras (Outras doenças glomerulares: amiloidose renal, gamopatia monoclonal de significado renal, glomerulonefrite associada à infecção, biópsia normal com hematúria e/ou proteinúria subnefrótica, glomerulonefrite fibrilar, glomerulonefrite mesangial não IgA, glomerulonefrite crescêntica não classificada nas doenças glomerulares anteriores, microangiopatia trombótica, sem diagnóstico histológico definido); Não biopsiados (sem biópsia renal); TFGe (taxa de filtração glomerular estimada); DP (desvio padrão); % (porcentagem); IIQ (intervalo interquartil); < (menor); = (maior ou igual); FIAT (fibrose intersticial e atrofia tubular); DRC (doença renal crônica).

### Comparação de Diagnósticos Histológicos e Casos não Biopsiados

Os pacientes com NL apresentaram a menor média de idade, ao passo que os pacientes com glomerulone­frite membranoproliferativa (GNMP) apresentaram a maior. A NL foi mais frequente no sexo feminino, enquanto a vasculite associada a ANCA (ANCAV) e a NM predominaram no sexo masculino. Todos os casos de ANCAV foram observados em pacientes brancos (Tabela 1).

A GNMP esteve associada a 100% de prevalência de HAS, enquanto a doença de lesão mínima (DLM) apresentou a menor prevalência de hipertensos (63,3%). Os transtornos psiquiátricos foram mais comuns em pacientes com DLM, e os pacientes com GNMP registraram a maior prevalência de tabagismo. As DCVs foram mais frequentes em pacientes com GNMP e GESF (Tabela 1). A GNMP esteve associada a níveis iniciais significativamente mais elevados de PAS (p < 0,001) e PAD (p = 0,004) (Tabela 1).

O uso de medicamentos variou entre os grupos: os antiproteinúricos foram utilizados por todos os pacientes com DLM e GESF e por 71,3% dos casos não biopsiados (p < 0,001). Os diuréticos foram prescritos com maior frequência para pacientes com GNMP (100%), DLM (93,3%), NM (92%) e GESF (84,6%) (p < 0,001). As estatinas foram prescritas com maior frequência em ANCAV (90,9%) e NM (88%) (p < 0,001), ao passo que os inibidores de SGLT2 foram mais comumente utilizados em pacientes com NIgA (31,4%) (p < 0,001) (Tabela 1).

A terapia imunossupressora foi administrada em todos os casos de NL e ANCAV e foi também prevalente na DLM (93,3%) e na NM (72,9%) (p < 0,001). A SN predominou em pacientes com DLM (96,7%), NM (92%) e GESF (53,8%). A GNRP foi mais frequente em ANCAV (60,0%) e NL (20,3%), por outro lado nenhum caso foi observado em DLM ou NM (p < 0,001). A ANCAV apresentou a maior necessidade de diálise (30,0%) (p < 0,001) (Tabela 1).

A análise histopatológica revelou mais crescentes nos casos de ANCAV (100%) e NL (45,5%) (p < 0,001), enquanto a FIAT foi mais comum em GNMP (88,9%), NIgA (80%) e GESF (79,1%) (p < 0,001). A esclerose glomerular (total ou segmentar, excluindo a GESF) foi prevalente nos casos de NIgA (88,1%) e ANCAV (85,7%), porém menos frequente nos casos de DLM (46,7%) (p < 0,001) (Tabela 2).

### Desfechos

Durante o acompanhamento, a TFGe média diminuiu de 72,9 ± 33,3 para 62,5 ± 33,0 (p < 0,001), ao passo que a proteinúria diminuiu de 3,7 ± 4,0 para 1,3 ± 2,1 (p < 0,001) (Tabela 2). A DLM apresentou a melhor sobrevida renal, enquanto a GNMP apresentou os piores desfechos (Figura 4). A maioria dos pacientes (61,4%) permaneceu em acompanhamento, com baixas taxas de TRS crônica (4,6%) ou mortalidade (1,2%) (Tabela 2).

**Figura 4:** Curva de sobrevida renal (Kaplan-Meier) na comparação dos principais diagnósticos das doenças glomerulares (log rank 0,001).

Abreviações – NL (Nefrite Lúpica), NIgA (Nefropatia por IgA), NM (Nefropatia Membranosa), GESF (Glomeruloesclerose Segmentar e Focal), DLM (Doença de Lesão Mínima), GNMP (Glomerulonefrite Membranoproliferativa), ANCAV (Vasculite associada a ANCA), Outras (Outras DG: amiloidose renal; gamopatia monoclonal de significado renal; glomerulonefrite associada à infecção; biópsia normal com hematúria e/ou proteinúria subnefrótica; glomerulonefrite fibrilar; glomerulonefrite mesangial não IgA; glomerulonefrite crescêntica não classificada em DG anteriores; microangiopatia trombótica; sem diagnóstico histológico definido), Sem biópsia renal (Sem biópsia).

A análise de regressão de Cox identificou fatores de proteção para a progressão da DRC, tais como a TFGe basal mais elevada (HR = 0,956, IC95%: 0,920–0,994; p = 0,023). Os fatores de risco para a progressão da DRC incluíram a presença de FIAT (HR = 1,079, IC95%: 1,020–1,142; p = 0,008) e IMC mais alto (HR = 1,257, IC95%: 1,016–1,556; p = 0,035) (Tabela 3).

**Tabela 3:** Análise de regressão de cox

Análise de regressão de Cox: desfechos de sobrevida renal ajustados para fatores de confusão			
	HR	IC 95%	*p*
Idade ao diagnóstico de DG	1,041	0,955–1,134	0,366
Sexo feminino	0,163	0,022–1,211	0,076
TFGe inicial	0,956	0,920–0,994	**0,023**
Proteinúria inicial	0,932	0,687–1,263	0,648
DM (diagnóstico presente)	0,985	0,153–6,348	0,988
IMC inicial	1,257	1,016–1,556	**0,035**
FIAT	1,079	1,020–1,142	**0,008**
Inibidores da ECA ou bloqueadores do receptor de angiotensina (uso)	83,3	0,420–16502,754	0,101
Imunossupressão (uso)	0,626	0,080–4,881	0,655

Legenda: DG: doença glomerular; TFGe: taxa de filtração glomerular estimada; DM: diabetes mellitus; IMC: índice de massa corporal; FIAT: fibrose intersticial e atrofia tubular; ECA: enzima conversora de angiotensina; HR: *Hazard ratio*; IC: Intervalo de confiança.

Com o tempo, a prevalência de casos de NL aumentou, enquanto as podocitopatias (DLM e GESF) apresentaram redução e a NIgA permaneceu estável (Figura 5).

**Figura 5:** Evolução temporal da prevalência dos principais diagnósticos histopatológicos das doenças glomerulares de Janeiro de 1998 a Janeiro de 2023.

Abreviações – NL (Nefrite Lúpica), NIgA (Nefropatia por IgA), NM (Nefropatia Membranosa), GESF (Glomeruloesclerose Segmentar e Focal), DLM (Doença de Lesão Mínima), GNMP (Glomerulonefrite Membranoproliferativa), ANCAV (Vasculite associada a ANCA).

## Discussão

Nosso estudo confirmou uma mudança no perfil epidemiológico das DG na região ao longo dos últimos 25 anos. Além disso, proporcionou novas percepções ao incorporar dados sobre desfechos renais, juntamente com taxas de prevalência e descrições de diagnósticos histopatológicos.

Um estudo multinacional que analisou a frequência de DG na Europa, América do Norte, América Latina e Ásia relatou uma mediana de idade de 47,3 anos (39,7–56,9), com 53% dos pacientes do sexo masculino e 57% identificando-se como brancos^10^. Os pacientes da América Latina eram mais jovens e predominantemente do sexo feminino, em consonância com nossos achados. Esse perfil demográfico, caracterizado por uma predominância de pacientes jovens do sexo feminino, provavelmente está relacionado à alta prevalência de NL na América Latina e no Brasil, com sugestão de possível componente genético^21^.

Sobre esse aspecto, uma proporção significativa de pacientes (23,3%) relatou histórico familiar de nefropatia, o que reforça a influência genética nas DG^22^. Um estudo realizado em 163 pacientes com DRC de etiologia desconhecida (2016–2020) identificou um diagnóstico genético definitivo em 31% dos 100 pacientes com DG^23^. O sequenciamento do exoma de 111 casos de SN idiopática não revelou variantes patogênicas no grupo sensível a corticosteroides, enquanto 30% dos casos resistentes a corticosteroides abrigaram variantes patogênicas em genes associados a podocitopatias^24^.

Essa população apresentou um alto risco cardiovascular, com comorbidades e eventos clínicos significativos, em consonância com estudos anteriores que ressaltam a conexão entre fatores de risco cardiovascular tradicionais e fatores específicos da DG, nos quais se inclui seu tratamento^25,26^. Um estudo de coorte com 1.912 pacientes com DGP (NIgA, GESF, DLM ou NM) constatou que a taxa de eventos cardiovasculares nessa população foi 2,5 vezes maior do que na população geral, e os pacientes com GESF apresentaram o maior risco^25^. Uma coorte dinamarquesa de base populacional com 1.644 pacientes com lúpus eritematoso sistêmico (LES) identificou um risco significativamente maior de infarto agudo do miocárdio (IAM) (aumento de oito vezes) e mortalidade cardiovascular (aumento de quatro vezes) entre pacientes com LES com NL em comparação com aqueles sem NL^26^.

Além disso, os transtornos psiquiátricos foram mais frequentes em subgrupos de doenças que demandaram maior uso de corticosteroides - a principal terapia imunossupressora em nosso estudo – o que reforça uma associação bem estabelecida^27^. Em estudo de caso-controle que comparou 50 pacientes com LES e NL, 50 pacientes com LES sem NL e 50 controles saudáveis, foi constatada maior prevalência de ansiedade e depressão entre os pacientes com NL, com risco aumentado naqueles de idade avançada e com maior atividade histológica^28^. Outro estudo que investigou a associação entre transtornos psiquiátricos e DGP entre 950 participantes relatou uma prevalência de 12% de diagnósticos psiquiátricos. Esse diagnóstico foi significativamente associado à progressão da DRC (HR = 2,45, IC 95%: 1,53–3,92)^29^.

O Brasil é um país de dimensões continentais e, como tal, são esperadas variações regionais nos padrões das DG. Segundo dados do Registro Pernambucano de Glomerulopatias de 2017, a região Nordeste relatou que a GESF (43,0%) foi a DGP mais comum, enquanto a NL (67,0%) foi a DGS predominante^12^. Um estudo realizado em 2015 no Ceará encontrou proteinúria na faixa nefrótica em 67,3% dos pacientes, e os diagnósticos histopatológicos mais frequentes foram GESF (19,6%), DLM (17,9%), NM (16,7%) e NL (11,9%)^13^. Da mesma forma, um levantamento realizado em 2016 no sul do Brasil encontrou uma maior prevalência de doenças glomerulares secundárias (61,2%), e a NL (30,2%) o diagnóstico mais comum. Entre as DGP, a GESF permaneceu como a mais frequente (10,3%)^14^. Um estudo de 2010 do Distrito Federal relatou a GESF (26,9%) como a DGP mais prevalente, enquanto a NL (50,0%) foi a principal DGS^15^. No norte do Brasil (estado do Amazonas), a GESF também foi identificada como a DG mais comum^16^. Uma análise de cinco anos de casos de DG em São Paulo, publicada no Registro Paulista de Glomerulopatias de 2006, foi realizada com dados de um estudo multicêntrico envolvendo 11 centros que analisaram 1.844 biópsias renais. As DGP mais frequentes foram a GESF (29,7%), a NM (20,7%) e a NIgA (17,8%), ao passo que a NL foi a DGS mais comum (66,2%)^17^. O primeiro relatório do Registro Brasileiro de Biópsia Renal foi publicado recentemente. O estudo avaliou 954 biópsias e representou todas as regiões brasileiras. O principal diagnóstico de DG foi NL (22,6%), seguido por NIgA (13%) e GESF (12,2%)^30^.

Em nosso estudo, a NL manteve-se como a DGS mais prevalente em nossa região, em consonância com os achados de um estudo internacional de 2018 e de outros estudos brasileiros^10–18^. No entanto, na análise das DGP, a GESF foi o terceiro diagnóstico mais comum, juntamente com a DLM, atrás da NM e da NIgA. Isso contrasta com estudos anteriores que identificaram a GESF como a DGP mais frequente^11–18^. Esse achado reflete os avanços na compreensão da fisiopatologia das DG, reconhecendo a GESF não como uma simples doença, mas como um padrão histopatológico de lesão glomerular associado a múltiplos mecanismos patológicos que têm como alvo principal o podócito^31^.

Com relação a outros achados histopatológicos, nossa análise revelou um perfil sugestivo de cronicidade avançada, o que destaca a necessidade de indicações mais precoces de biópsia renal, mesmo em casos de hematúria glomerular persistente e/ou proteinúria com níveis mais baixos. Um estudo japonês com 552.951 participantes encontrou hematúria em 26,5%, proteinúria ocasional em 10,1% e proteinúria persistente em 1,5%. Além disso, um declínio na TFGe foi associado tanto à hematúria isolada quanto ao seu efeito sinérgico com a proteinúria^32^.

Ao longo do acompanhamento, a TFGe diminuiu, enquanto os níveis de proteinúria apresentaram melhora. Esses achados provavelmente refletem a progressão da doença, com uma redução da TFGe ao longo do tempo, e os efeitos do tratamento - tanto imunossupressores quanto não imunossupressores – na evolução da proteinúria. Indicadores adicionais de desfechos favoráveis incluem que a maioria dos pacientes permanece em acompanhamento ativo, com baixas taxas de progressão para TRS e/ou mortalidade.

A sobrevida renal foi mais elevada na DLM e mais baixa na GNMP. Em nosso estudo, os pacientes com DLM eram mais jovens, apresentaram escores mais baixos de FIAT e de lesões vasculares e exibiram valores mais altos de TFGe inicial e final. Eles também apresentaram maiores reduções na proteinúria, sugerindo uma resposta terapêutica positiva e remissão da doença. Um estudo de análise de risco sobre a progressão da DRC, necessidade de TRS, eventos cardiovasculares e mortalidade em 907 adultos com SN primária (DLM, GESF ou NM), em comparação com um grupo controle saudável, identificou riscos menores em pacientes com DLM, exceto para tromboembolismo venoso^33^. Os pacientes com GNMP em nosso estudo apresentaram a maior média de idade, indicadores histológicos sugestivos de cronicidade, níveis mais baixos de TFGe inicial e final e melhora mínima na proteinúria, o que indica uma pior resposta terapêutica. Um estudo brasileiro que analisou 53 casos de GNMP relatou remissão parcial ou completa em apenas 39% dos pacientes^34^.

A sobrevida renal em casos não biopsiados foi semelhante à observada na NIgA. Um estudo recente encontrou piores desfechos em casos de NIgA e GESF, com taxas mais elevadas de progressão para TRS^35^. Esses achados reforçam ainda mais a necessidade de reconsiderar as indicações de biópsia renal para anormalidades urinárias persistentes que, tradicionalmente, podem não justificar a avaliação histopatológica, bem como a importância de expandir as análises genéticas^22,23^.

Os principais fatores associados à progressão da DRC foram a TFGe inicial mais baixa, a presença de FIAT e o IMC mais alto. A associação entre menor TFGe basal e piores desfechos pode refletir a seleção de casos com maior gravidade clínica e/ou histopatológica no início da doença. Um estudo coreano que analisou 1.943 biópsias renais de pacientes com DGP constatou uma pior progressão da doença naqueles com TFGe abaixo de 60 mL/min/1,73m^2 36^. Além disso, a gravidade da FIAT é um achado classicamente associado à cronicidade e a desfechos desfavoráveis^37^. A obesidade é um fator de risco bem estabelecido para DRC e tem sido especificamente relacionada às glomerulopatias^38^. Uma revisão sistemática e meta-análise de casos de NIgA constatou que os pacientes com sobrepeso e obesidade apresentavam TFGe mais baixa em comparação com aqueles com IMC normal^39^. Um estudo japonês que analisou 489 pacientes com NL identificou o IMC e a TFGe basal como fatores de risco independentes para a progressão da DRC^40^.

As principais limitações deste estudo incluíram o período de análise prolongado, que levou à perda de acompanhamento de alguns pacientes, e restrições na coleta de dados devido ao seu delineamento retrospectivo, uma vez que se baseou exclusivamente em registros médicos. Semelhante à maioria dos estudos sobre esse tema, nossa investigação concentrou-se nos padrões histopatológicos das lesões glomerulares, e não em sua fisiopatologia subjacente^10–18^. A próxima etapa lógica é aprofundar a avaliação de cada subgrupo de lesões, inclusive daquelas que não foram submetidas à análise histológica. Esse aprofundamento deve combinar métodos descritivos, analíticos e inferenciais, integrados a novas abordagens clínicas, laboratoriais e genéticas.

Em conclusão, a NL permaneceu como a DGS mais prevalente em nossa região, enquanto a GESF e a DLM ocuparam o terceiro lugar entre as DGP, após a NM e a NIgA. A sobrevida renal foi mais elevada na DLM e mais baixa na GNMP. A compreensão do perfil epidemiológico das DG em nosso meio é fundamental para o desenvolvimento de políticas de saúde voltadas ao cuidado integral desse grupo específico de pacientes com doença renal.

## Data Availability

Os dados serão compartilhados mediante solicitação razoável ao autor correspondente.
